# The Exponential Phase of the Covid-19 Pandemic in Central Italy: An Integrated Care Pathway

**DOI:** 10.3390/ijerph17113792

**Published:** 2020-05-27

**Authors:** Carlo Capalbo, Antonio Aceti, Maurizio Simmaco, Rita Bonfini, Monica Rocco, Alberto Ricci, Christian Napoli, Matteo Rocco, Valeria Alfonsi, Antonella Teggi, Giovanni Battista Orsi, Marina Borro, Iolanda Santino, Robert Preissner, Paolo Marchetti, Adriano Marcolongo, Paolo Anibaldi

**Affiliations:** 1 Medical Oncology Unit Sant’Andrea Hospital, University La Sapienza, 00189 Rome, Italy; carlo.capalbo@uniroma1.it (C.C.); paolo.marchetti@uniroma1.it (P.M.); 2 Department of Molecular Medicine, University La Sapienza, 00189 Rome, Italy; 3 Infectious Diseases Unit Sant’Andrea Hospital, University La Sapienza, 00189 Rome, Italy; antonio.aceti@uniroma1.it (A.A.); ateggi@ospedalesantandrea.it (A.T.); 4 Laboratory Analysis and Advanced Molecular Diagnostics Unit, Sant’Andrea Hospital, Sapienza University of Rome, 00189 Rome, Italy; maurizio.simmaco@uniroma1.it (M.S.); marina.borro@uniroma1.it (M.B.); 5 Emergency Department Unit Sant’Andrea Hospital, University La Sapienza, 00189 Rome, Italy; rbonfini@ospedalesantandrea.it; 6 Anesthesia and Intensive Care Medicine, Department of Clinical and Surgical Translational Medicine, Sant’Andrea Hospital, Sapienza University of Rome, 00189 Rome, Italy; mrocco@ospedalesantandrea.it; 7 Division of Pneumology, Department of Clinical and Molecular Medicine, Sapienza University of Rome, AOU Sant’Andrea, 00189 Rome, Italy; alberto.ricci@uniroma1.it; 8 Medical Direction Sant’Andrea Hospital, University La Sapienza, 00189 Rome, Italy; christian.napoli@uniroma1.it (C.N.); mrocco@ospedalesantandrea.it (M.R.); valfonsi@ospedalesantandrea.it (V.A.); 9 Department of Public Health and Infectious Diseases, University La Sapienza, 00189 Rome, Italy; giovanni.orsi@uniroma1.it; 10 Microbiology Unitand Advanced Molecular Diagnostics Unit, Sant’Andrea Hospital, Sapienza University of Rome, 00189 Rome, Italy; iolanda.santino@uniroma1.it; 11 Department of Physiology, CharitéUniversitätsmedizin Berlin, Charitéplatz 1, D-10117 Berlin, Germany; robert.preissner@charite.de; 12 General Direction Sant’Andrea Hospital, University La Sapienza, 00189 Rome, Italy; amarcolongo@ospedalesantandrea.it

**Keywords:** coronavirus, COVID-19, clinical pathway, Italy, pandemic, SARS-Cov-2, risk management

## Abstract

The Coronavirus Disease (Covid-19) pandemic is rapidly spreading across the world, representing an unparalleled challenge for health care systems. There are differences in the estimated fatality rates, which cannot be explained easily. In Italy, the estimated case fatality rate was 12.7% in mid-April, while Germany remained at 1.8%. Moreover, it is to be noted that different areas of Italy have very different lethality rates. Due to the complexity of Covid-19 patient management, it is of paramount importance to develop a well-defined clinical workflow in order to avoid the inconsistent management of patients. The Integrated Care Pathway (ICP) represents a multidisciplinary outline of anticipated care to support patient management in the Sant’Andrea Hospital, Rome. The main objective of this pilot study was to develop a new ICP evaluated by care indicators, in order to improve the COVID-19 patient management. The suggested ICP was developed by a multi-professional team composed of different specialists and administrators already involved in clinical and management processes. After a review of current internal practices and published evidences, we identified (1) the activities performed during care delivery, (2) the responsibilities for these activities, (3) hospital structural adaptation needs and potential improvements, and (4) ICP indicators. The process map formed the basis of the final ICP document; 160 COVID-19 inpatients were considered, and the effect of the ICP implementation was evaluated over time during the exponential phase of the COVID-19 pandemic. In conclusion, a rapid adoption of ICP and regular audits of quality indicators for the management of COVID-19 patients might be important tools to improve the quality of care and outcomes.

## 1. Introduction

The Coronavirus Disease pandemic is rapidly spreading across the world, and it is an unparalleled challenge for the medical community and national health systems. Severe Acute Respiratory Syndrome Coronavirus 2 (SARS-CoV-2) infection is a very insidious pathology, and symptomatic manifestations can range from mild to critical. Some patients with initially mild symptoms may quickly progress over the course of five to ten days. In particular, earlier studies on a hospitalized Chinese population for pneumonia due to SARS-CoV-2 showed that dyspnea developed after a median of five days from the onset of symptoms, and hospital admission occurred after a median of seven days of symptoms [1,2]. Recovery time appears to be around two weeks for mild infections and three to six weeks for severe disease [3]. In Italy, 12%–16% of all hospitalized patients were admitted to the intensive care unit, and the estimated case fatality rate was 12.7% in mid-April [4,5,6]. This value is considerably different from that reported in South Korea (about 2%) even if the outbreak started concomitantly in the two countries [7]. This, in part, may be related to different impacts such as political management of the health crisis, availability of ventilators, and demographic factors (e.g., different median age of patients) [5]. It appears less comprehensible how different areas of Italy have very different lethality rates. In particular, the estimated case fatality rate in Lazio (Central Italy) is lower (5.7%) than in other central regions (e.g., Marche: 13.4%) and much lower than in Northern Italy (Lombardy: 18.4%) [7].

Even if a part of these differences can be explained by the pandemic “surprise effect” in Northern Italy, where the pandemic started, as well as to regional differences in socio-demographic conditions, it is noteworthy that the Italian National Health Service (INHS) is organized on a regional basis and that each Italian Region has large autonomy in health services management. This circumstance and its impact on the local intensity and severity of COVID-19 deserves in-depth analysis, also considering the significant difference in epidemic outcomes observed between Lombardy and Veneto, two neighboring regions with similar socio-economic profiles and with similar timing in the pandemic rise. The Governors of Lombardy and Veneto made different management choices for COVID-19 cases, and indeed, a re-arrangement of the INHS based on the regional pandemic experiences is becoming a debated political and social issue in Italy.

Particularly, it is becoming increasingly apparent that different health management choices made early in the cycle of the pandemic had a relevant impact. Besides that, due to the magnitude and the complexity of COVID-19 management, it is of paramount importance to develop structured care in order to avoid additional irrational and inconsistent management of patients. To contribute to the improvement of present and future pandemic management, here we report the experience of the Sant’Andrea University Hospital of Rome (Region Lazio, Central Italy). In Lazio, the regional hospital network for COVID-19 management was early divided into hub-and-spoke structures, and Sant’Andrea University Hospital is one out of five hubs in Rome. To date, about 70% of all SARS-CoV-2-positive patients in Lazio are treated in Rome [8]. We established a multi-disciplinary team to develop and implement an Integrated Care Pathway (ICP) for COVID-19 patients in order to improve the standard of care and minimize infection diffusion. We also report a preliminary assessment of the ICP impact by care indicators.

## 2. Materials and Methods 

A multi-professional team, composed of different types of physicians and administrators, who manage both clinical and organizational processes and are responsible for patient care, was created. The team includes: infectious disease specialists, hospital pharmacist, health director, oncologist, pneumologist, radiologist, laboratory medicine specialist, health administrators, internal medicine specialist, public health specialist, intensive care specialist. A facilitator to act as a link between all experts was also designated. Every decision was achieved with a majority of votes. The first steps of ICP development were a rapid review of both the current internal practice and the published evidences on COVID-19 management. During this process, selected literature was thoroughly screened for identification of all possible recommendations by the members of the working group; moreover, participants were given the opportunity to produce specific recommendations as expert opinions in the absence of evidence of effectiveness or indication in available guidelines. In particular, the specific COVID-19 documents produced by SIMIT (Italian Society for Infectious and Tropical Diseases), AIFA (Italian Medicines Agency), and EMA (European Medicines Agency) were taken into consideration. Evidence Definitions: Class I: Conditions for which there is evidence, general agreement, or both that a given procedure or treatment is useful and effective; Class II: Conditions for which there is conflicting evidence, a divergence of opinion, or both about the usefulness/efficacy of a procedure or treatment; Class IIa: Weight of evidence/opinion is in favor of usefulness/efficacy. Class IIb: Usefulness/efficacy is less well established by evidence/opinion. Class III: Conditions for which there is evidence, general agreement, or both that the procedure/treatment is not useful/effective and in some cases may be harmful. Level of Evidence A: Data derived from multiple randomized clinical trials. Level of Evidence B: Data derived from a single randomized trial or nonrandomized studies. Level of Evidence C: Consensus opinion of experts. Then, to define the process map for COVID-19, we also identified: (i) the activities performed during the delivery of care to the patients; (ii) the responsibilities for these activities; (iii) hospital structural adaptation needs; and (iv) potential problem areas or opportunities for improvements. The ICP was periodically improved according to the available updates. The impact of the developed ICP was assessed using the hospital fatality risk formula (fatal cases)/(fatal cases + recovered cases) as a care indicator, which provides a more accurate early estimate compared with (fatal cases)/(all cases). Statistical analysis was performed using the SPSS statistics software version 20 (IBM Corp, New York, NY, USA). With regard to ICP development, the study was performed in accordance with the World Medical Association Declaration of Helsinki and did not include any identifiable human data. For the following phase of patient enrolment, the protocol was approved by the Ethical committee of Sapienza University of Rome (reference number 5773_2020).

## 3. Results

### 3.1. Integrated Care Pathway 

**Figure 1:**
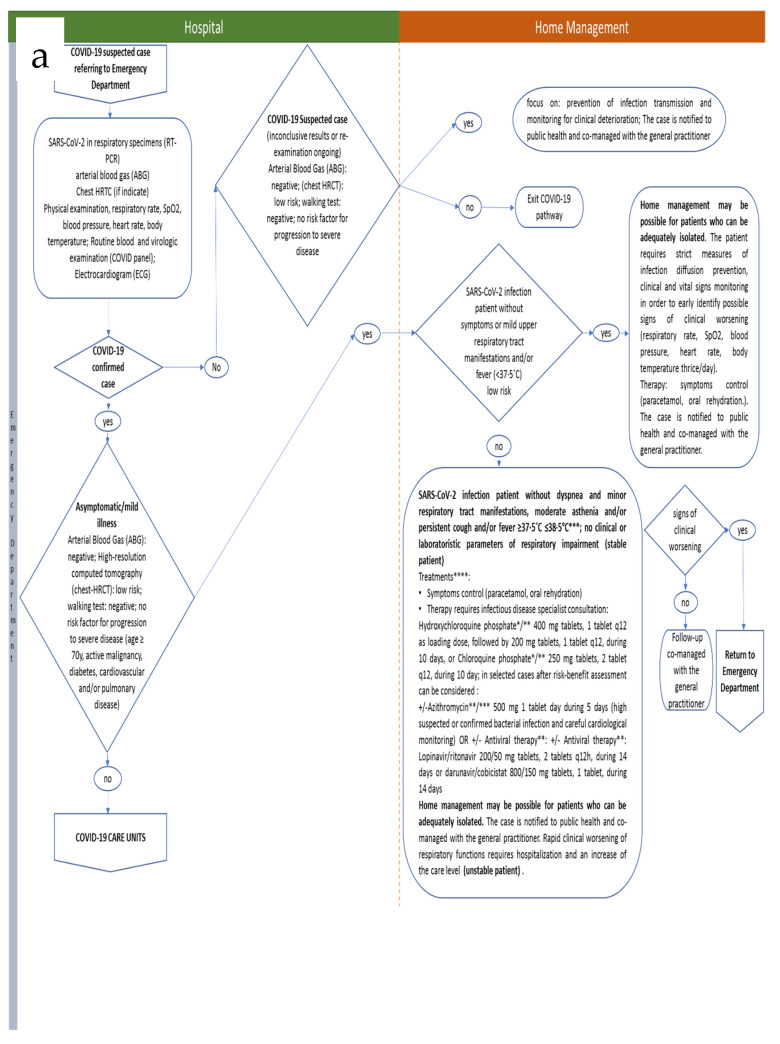

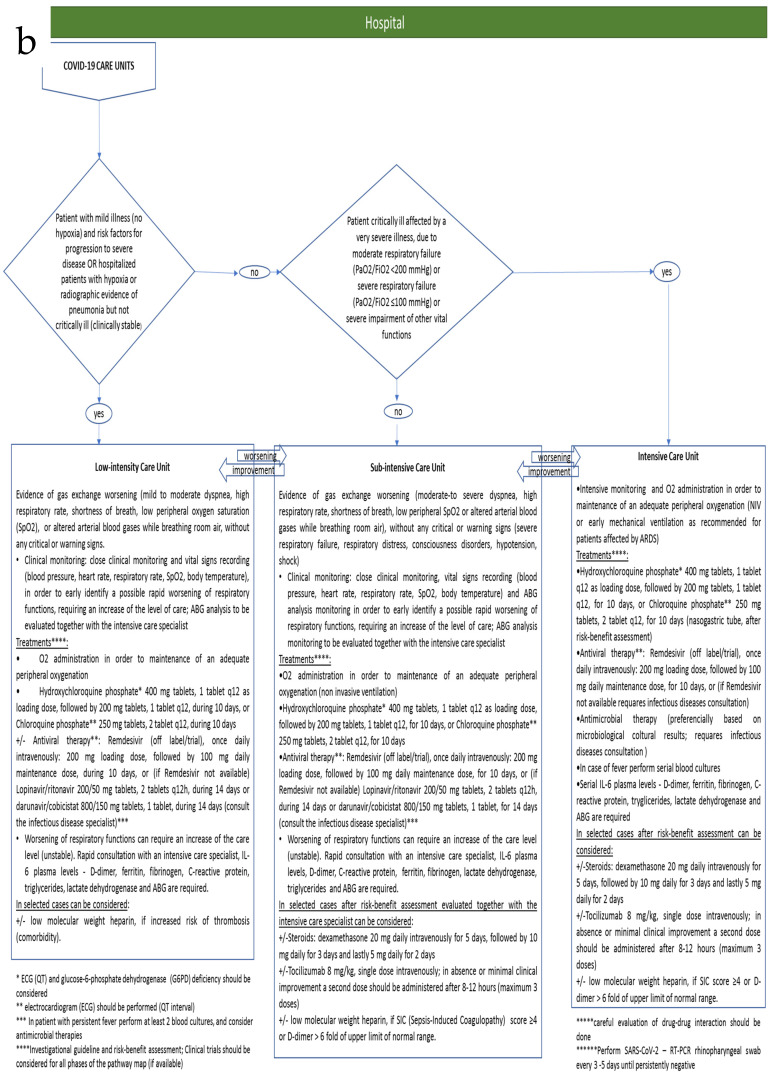
(**a**) Sant’Andrea Hospital Integrated Care Pathway (Hospital/Home). (**b)** Sant’Andrea Hospital Integrated Care Pathway (Hospital Care Units).

The process map for the developed ICP is reported in detail in Table 1 and Figure 1. It refers to the clinical management of adult, non-pregnant SARS-CoV-2 positive patients (as detected by real-time PCR analysis from naso-pharingeal swabs). All cases were notified to public health officials. To date, there are no solid data on drugs or other therapeutics to prevent or treat COVID-19. The clinical actions are based on clinical signs and severity of symptoms. Generally, management of most patients focuses on prevention of transmission to others and monitoring for clinical deterioration, to minimize and optimize hospitalization rate. Patients with mild illness who can be adequately isolated in the outpatient setting are directed to home management. For patients requiring hospitalization, the treatments mainly consist of supportive care, although for severe cases several investigational approaches are considered. In such cases, evaluation of drug–drug interactions of investigational COVID-19 therapies with co-medications is carried out to optimize patient’s specific therapy, avoiding predictable inefficacy/toxicity. For patients referred to the Emergency Department, interactions of investigational COVID-19 therapies with other drugs are evaluated using the interaction charts from Liverpool University [9,10]. For patients in low-intensity, sub-intensive, and intensive care units, drug–drug interactions are evaluated also using the Drug-PIN software, a clinical decision support system aimed at the patient-specific design of poly-pharmacy [11]. Clinical trials enrollment, if available, is considered for all phases of the process map. The patient enters the process at the Emergency Department, as the first switching point in the ICP is the definition as “Suspected COVID-19 case” or “Confirmed COVID-19 case”. “Suspected COVID-19 case” is defined as follows: a patient with acute respiratory illness (fever and at least one sign/symptom of respiratory disease, e.g., cough, shortness of breath) AND a history of travel to or residence in a location reporting community transmission of COVID-19 disease during 14 days prior to symptom onset OR a patient with any acute respiratory illness AND having been in contact with a confirmed or probable COVID-19 case in the last 14 days prior to symptom onset OR a patient with severe acute respiratory illness (fever and at least one sign/symptom of respiratory disease, e.g., cough, shortness of breath; and requiring hospitalization) and in the absence of an alternative diagnosis that fully explains the clinical presentation (according to WHO COVID-19 suspected case definition); suspected cases with inconclusive or negative results from RT-PCR for SARS-CoV-2 (discrepancy with clinical data) are tested on a further sample 24 h later. Confirmed COVID-19 case is defined as follows: a patient with laboratory confirmation of RT-PCR SARS-CoV-2 infection, irrespective of clinical signs and symptoms. Confirmed cases are differentially managed according to disease severity, as detailed in Table 1 and Figure 1a,b.

### 3.2. Preliminary Assessment of the Integrated Care Pathway

Between 8th March and 7th April 2020, 627 COVID-19 suspect adult patients were evaluated in our center. Out of 315 SARS-Cov-2 RNA-positive patients, 160 required hospitalization. Of them, 22 died during the period considered in the study. The median age of hospitalized died patients was 85 years. Most of them were male, and all were older than 50 years. Comorbidities were present in almost all the cohort, with hypertension being the most common comorbidity. We assessed the impact of the ICP on COVID-19 inpatients mortality by hospital fatality risk (HFR) as the indicator. As target values for HFR are not broadly available at present, we calculated the HFR from the retrospective cohort study including adult inpatients (≥18 years old) with laboratory confirmed COVID-19 from Jinyintan Hospital and Wuhan Pulmonary Hospital (Wuhan, China) during a well-defined period of time (29th December 2019–31st January 2020). The HFR value in this reference cohort was 22% [12]. As shown in Table 2, along the period of ICP application, the Sant’Andrea University Hospital HFR was 12.1%, significantly lower compared with the reference HFR [1,13,14,15].

## 4. Discussion

About three months after the COVID-19 pneumonia outbreak in Wuhan, Italy was affected by the SARS-Cov-2 pandemic. The epidemiologic differences we are noticing across the affected countries surely highlight different distribution of risk factors such as demographics, comorbidities, and many other features, making the comparison of data difficult. However, it becomes increasingly evident that different organization of health systems and different health policies, as well as the timeline of health management choices, have a great impact on the pandemic incidence. Comparative effectiveness research on different systems would provide precious information to develop better organizational models to face the pandemic. Thus, it is important to share methods and outcomes. The Italian paradigm may be an example, since in a generally similar social and demographic background, important differences in the pandemic management arose among different regions, leading to sensible differences in care outcomes. We developed an ICP which allowed to manage the suspected and confirmed COVID-19 cases referred to the emergency department, maintaining high quality standards and reducing the hospital fatality rate within acceptable limits. The availability of the ICP has been crucial to make early and shared decisions, to organize rationale triage areas with clear and definite protocols for triage, leading to testing suspect cases rapidly, and, depending on the diagnosis, to allocating them to the appropriate ward without delay. Thus, early and appropriate treatment of symptomatic patients has been possible together with strengthening of home management.

In particular, the Sant’Andrea University Hospital HFR is significantly lower than the Wuhan hospitals compressive data (Table 2) and a large series of hospitalized patients in the New York City Area, while, HFR is similar to that reported in a recent update study from a single hyper-specialized COVID Hospital in Wuhan (Wuhan Pulmonary Hospital) [12,13,14,15,16,17]. This observation suggests that the ICP here reported is suitable to allow a rapid and high-quality response also in relatively small health structures not specifically devoted to infective disease treatment. This is relevant to ensure an appropriate reaction of the INHS to the next phases of the pandemic, which could also present a further peak following removal of the lockdown. The ICP adoption during a well-defined interval of time in our emergency department resulted in a remarkable reduction of COVID-19-related mortality (before 60% vs. after 23.2%, data not shown).

## 5. Conclusions

Our findings suggest that a rapid adoption of ICP and regular audit of quality indicators for the management of COVID-19 patients might be important tools to improve the quality of care and outcomes.

## Figures and Tables

**Table 1:** Integrated Care Pathway for patient management

Action Area	Indication (To Support Clinician Activities)
Home Management (Asymptomatic Or Mild Infection)	SARS-CoV-2 infection patient without symptoms or mild upper respiratory tract manifestations and/or fever (<37.5 °C) and/or minor clinical manifestation (malaise, muscle pain, nasal congestion, or headache); arterial blood gas (ABG): negative; high-resolution computed tomography (chest-HRCT): low risk; walking test: negative; no risk factor for progression to severe disease (age ≥ 70 y, active malignancy, diabetes, cardiovascular and/or pulmonary disease). Team leader: emergency physician (Responsible); infectious disease specialist and pneumologist (Involved). Management of most patients should focus on prevention of transmission to others and monitoring for clinical deterioration, which should minimize hospitalization. Home management may be possible for patients with mild illness who can be adequately isolated The patient requires strict measures of infection diffusion prevention and clinical and vital signs monitoring in order to early identify possible signs of clinical worsening (respiratory rate, SpO2, blood pressure, heart rate, body temperature) thrice/day. The case is notified to public health and co-managed with the general practitioner Treatments: symptoms control (paracetamol, oral rehydration).
	SARS-CoV-2 infection patient without dyspnea and minor respiratory tract manifestations, moderate asthenia and/or persistent cough and/or fever ≥37.5 °C ≤ 38.5 °C ***; no clinical or laboratory parameters of respiratory impairment. Arterial blood gas (ABG): negative; high-resolution computed tomography (chest-HRCT): low risk; walking test: negative; no risk factor for progression to severe disease (age ≥ 70 y, active malignancy, diabetes, cardiovascular and/or pulmonary disease); stable patient. Team leader: emergency physician (Responsible); infectious disease specialist and pneumologist (Involved). Home management may be possible for patients with mild illness who can be adequately isolated. The patient requires strict measures of infection diffusion prevention, clinical and vital signs monitoring in order to early identify possible signs of clinical worsening (respiratory rate, SpO2, blood pressure, heart rate, body temperature) thrice/day. The case is notified to public health and co-managed with the general practitioner. Treatments: Symptoms control (paracetamol, oral rehydration). Therapy requires infectious disease specialist consultation: Hydroxychloroquine phosphate */** 400 mg tablets, 1 tablet q12 as loading dose, followed by 200 mg tablets, 1 tablet q12, for 10 days, or Chloroquine phosphate */** 250 mg tablets, 2 tablet q12, for 10 days; In selected cases after risk–benefit assessment can be considered: +/− Azitromicyn **/*** 500 mg 1 tablet day for 5 days (high suspected or confirmed bacterial infection and careful cardiological monitoring) or +/− Antiviral therapy **: Lopinavir/ritonavir 200/50 mg tablets, 2 tablets q12h, for 14 days or darunavir/cobicistat 800/150 mg tablets, 1 tablet, for 14 days; +/− low molecular weight heparin, (LMWH): consider if serious increased risk of thrombosis (comorbidity). Rapid worsening of respiratory functions requires hospitalization (unstable patient) and an increase of the care level. * ECG (QT interval) and glucose-6-phosphate dehydrogenase (G6PD) deficiency test should be considered ** ECG should be performed *** In patient with fever perform at least 2 blood cultures and consider antimicrobial therapies.
Hospitalized Patients Low-Intensity Care Unit	Hospitalized patients with mild illness (no hypoxia) and risk factors for progression to severe disease OR hospitalized patients with hypoxia or radiographic evidence of pneumonia but not critically ill (clinically stable). Team leader: infectious disease specialist (Responsible); internal medicine specialist and pneumologist (Involved). Evidence of gas exchange worsening (mild to moderate dyspnea, high respiratory rate, shortness of breath, low peripheral SpO2 or altered arterial blood gases while breathing room air), without any critical or warning signs. Clinical monitoring: close clinical monitoring and vital signs recording (blood pressure, heart rate, respiratory rate, SpO2, body temperature), in order to early identify a possible rapid worsening of respiratory functions, requiring an increase of the level of care; ABG analysis to be evaluated together with the intensive care specialist. Treatments: O2 administration in order to maintain adequate peripheral oxygenation; Hydroxychloroquine phosphate * 400 mg tablets, 1 tablet q12 as loading dose, followed by 200 mg tablets, 1 tablet q12, for 10 days, or Chloroquine phosphate ** 250 mg tablets, 2 tablet q12, for 10 days; In selected cases after risk–benefit assessment can be considered: +/− Antiviral therapy **: Remdesivir (off label/trial), once daily intravenously: 200 mg loading dose, followed by 100 mg daily maintenance dose, for 10 days, or (if Remdesivir not available) Lopinavir/ritonavir * 200/50 mg tablets, 2 tablets q12h, during 14 days or or Darunavir/Cobicistat 800/150 mg tablets, 1 tablet, for 14 days (requires infectious disease specialist consultation); +/− low molecular weight heparin (LMWH): consider if D-dimer > 6-fold of upper limit of normal range or increased risk of deep-vein thrombosis (comorbidity); +/− Antimicrobial therapy (preferentially based on microbiological culture results; requires infectious diseases specialist consultation); Worsening of respiratory functions can require an increase of the care level (unstable patient). Rapid consultation with an intensive care specialist, IL-6 plasma levels; D-dimer, ferritin, fibrinogen, C-reactive protein, triglycerides, lactate dehydrogenase, and ABG are required. * ECG (QT interval) and G6PD deficiency test should be considered ** ECG should be performed *** In patient with fever perform at least 2 blood cultures and consider antimicrobial therapies.
Hospitalized Patients Sub-Intensive Care Unit	Hospitalized patients with hypoxia or radiographic evidence of pneumonia but not critically ill (clinically stable) with need for high flow oxygen/Non Invasive Ventilation (NIV). Team leader: pneumologist (Responsible); infectious disease specialist (Involved). Evidence of gas exchange worsening (moderate-to-severe dyspnea, high respiratory rate, shortness of breath, low peripheral SpO2, or altered arterial blood gases while breathing room air), without any critical or warning signs (severe respiratory failure, respiratory distress, consciousness disorders, hypotension, shock). Clinical monitoring: close clinical monitoring, vital signs recording (blood pressure, heart rate, respiratory rate, SpO2, body temperature) and ABG analysis monitoring in order to early identify a possible rapid worsening of respiratory functions, requiring an increase of the care level; ABG analysis monitoring should be evaluated together with the intensive care specialist. Treatments: O2 administration in order to maintain adequate peripheral oxygenation (non-invasive ventilation); Hydroxychloroquine phosphate * 400 mg tablets, 1 tablet q12 as loading dose, followed by 200 mg tablets, 1 tablet q12, for 10 days, or Chloroquine phosphate ** 250 mg tablets, 2 tablet q12, for 10 days; Antiviral therapy **: Remdesivir (off label/trial), once daily intravenously: 200 mg loading dose, followed by 100 mg daily maintenance dose, for 10 days or (if Remdesivir not available) Lopinavir/ritonavir * 200/50 mg tablets, 2 tablets q12h, during 14 days or Darunavir/Cobicistat 800/150 mg tablets, 1 tablet, for 14 days (requires infectious disease specialist consultation); Worsening of respiratory functions can require an increase of the care level (unstable patient). Rapid consultation with an intensive care specialist, IL-6 plasma levels; D-dimer, ferritin, fibrinogen, C-reactive protein, triglycerides, lactate dehydrogenase, and ABG are required. In selected cases, after risk–benefit assessment together with the intensive care specialist, can be considered: +/− Steroids: dexamethasone 20 mg daily intravenously for 5 days, followed by 10 mg daily for 3 days and lastly 5 mg daily for 2 days; +/− Tocilizumab 8 mg/kg, single dose intravenously; in absence or minimal clinical improvement a second dose should be administered after 8–12 h (maximum 3 doses); +/− low molecular weight heparin, (LMWH): consider if SIC score ≥4 or D-dimer > 6-fold of upper limit of normal range or increased risk of deep-vein thrombosis (comorbidity); +/− Antimicrobial therapy (preferentially based on microbiological culture results; requires infectious diseases specialist consultation); * ECG and G6PD deficiency test should be considered ** ECG should be performed *** In patient with fever perform at least 2 blood cultures and consider antimicrobial therapies.
Hospitalized Patients Intensive Care Unit	Hospitalized patients critically ill affected by a very severe illness, due to moderate respiratory failure (paO2/FiO2 < 200 mmHg) or severe respiratory failure (PaO2/FiO2 < 100 mmHg) and/or severe impairment of other vital functions. Team leader: intensive care specialist (Responsible); infectious disease specialist (involved). Treatments: Intensive vital signs monitoring; O2 administration in order to maintain adequate peripheral oxygenation (NIV or early mechanical ventilation as recommended for patients affected by ARDS); Hydroxychloroquine phosphate * 400 mg tablets, 1 tablet q12 as loading dose, followed by 200 mg tablets, 1 tablet q12, for 10 days, or Chloroquine phosphate ** 250 mg tablets, 2 tablet q12, for 10 days (nasogastric tube), requires infectious diseases specialist consultation; Antiviral therapy **: Remdesivir (off label/trial), once daily intravenously: 200 mg loading dose, followed by 100 mg daily maintenance dose, for 10 days or if Remdesivir not available, requires infectious diseases specialist consultation; Antimicrobial therapy (preferentially based on microbiological culture results; requires infectious diseases specialist consultation); In case of fever perform serial blood cultures; Serial IL-6 plasma levels; D-dimer, ferritin, fibrinogen, C-reactive protein, triglycerides, lactate dehydrogenase, and ABG are required; In selected cases after risk–benefit assessment can be considered: +/− Steroids: dexamethasone 20 mg daily intravenously for 5 days, followed by 10 mg daily for 3 days and lastly 5 mg daily for 2 days +/− Tocilizumab 8 mg/kg, single dose intravenously; in absence or minimal clinical improvement a second dose should be administered after 8–12 h (maximum 3 doses) +/− low molecular weight heparin, (LMWH) consider if SIC score ≥ 4 or D-dimer > 6-fold of upper limit of normal range. * ECG and G6PD deficiency test should be considered ** ECG should be performed
	For all patients tested COVID positive, a psychological support was actively offered by psychologists trained for emergency management. The support was delivered 7 days/week from 8 am to 8 pm. In all not critically ill hospitalized patients perform SARS-CoV-2–RT-PCR rhinopharyngeal swab every 3–5 days until persistently negative. Therapy described within this document are based on very limited and preliminary clinical evidences which may be modified according to newly produced literature data. Clinicians must consider guidelines for investigational treatments, literature (or regulatory) updates, case-by-case indications/contraindications, and if available, clinical trials enrolment for all phases of the pathway map. It helped organize our internal critical discussions around COVID-19 clinical management.
	SpO2: peripheral oxygen saturation; ECG: electrocardiogram; G6PD: glucose-6-phosphate dehydrogenase; ABG: arterial blood gas; IL-6: Interleukine 6; SIC: sepsis-induced coagulopathy score

**Table 2:** Hospital fatality risk

Indicator	Hospital Fatality Risk (%)		*p*-Value **
	Sant’Andrea Hospital	Reference indicatorWuhan Hospitals	0.0253
(fatal cases)/(fatal cases + recovered cases)	22/182 (12.1%)	54/245 < 22% *	

* calculated from Fei Zhou et al. Lancet 2020; 395: 1054–62; ** chi-square statistic, *p* < 0.05.
